# Advancing the missed mutualist hypothesis, the under-appreciated twin of the enemy release hypothesis

**DOI:** 10.1098/rsbl.2022.0220

**Published:** 2022-10-19

**Authors:** Angela T. Moles, Rhiannon L. Dalrymple, S. Raghu, Stephen P. Bonser, Jeff Ollerton

**Affiliations:** ^1^ Evolution & Ecology Research Centre, School of Biological, Earth and Environmental Sciences, UNSW Sydney, Sydney, New South Wales 2052, Australia; ^2^ CSIRO, GPO Box 2583, Brisbane, Queensland 4001, Australia; ^3^ Faculty of Arts, Science and Technology, University of Northampton, Waterside Campus, Northampton NN1 5PH, UK; ^4^ Kunming Institute of Botany, Chinese Academy of Sciences, Kunming 650201, Yunnan, People's Republic of China

**Keywords:** enemy release hypothesis, introduced species, invasion, pollination, mutualism, seed dispersal

## Abstract

Introduced species often benefit from escaping their enemies when they are transported to a new range, an idea commonly expressed as the enemy release hypothesis. However, species might shed mutualists as well as enemies when they colonize a new range. Loss of mutualists might reduce the success of introduced populations, or even cause failure to establish. We provide the first quantitative synthesis testing this natural but often overlooked parallel of the enemy release hypothesis, which is known as the missed mutualist hypothesis. Meta-analysis showed that plants interact with 1.9 times more mutualist species, and have 2.3 times more interactions with mutualists per unit time in their native range than in their introduced range. Species may mitigate the negative effects of missed mutualists. For instance, selection arising from missed mutualists could cause introduced species to evolve either to facilitate interactions with a new suite of species or to exist without mutualisms. Just as enemy release can allow introduced populations to redirect energy from defence to growth, potentially evolving increased competitive ability, species that shift to strategies without mutualists may be able to reallocate energy from mutualism toward increased competitive ability or seed production. The missed mutualist hypothesis advances understanding of the selective forces and filters that act on plant species in the early stages of introduction and establishment and thus could inform the management of introduced species.

## 1. Introduction

The enemy release hypothesis is among the most prominent explanations for the success of introduced species [1]. This hypothesis predicts that species escape from some of their natural enemies during transport to and establishment in a new range, and the reduced effects of enemies allow introduced species to increase in abundance and distribution [2–4]. Many field studies have demonstrated that introduced populations suffer lower herbivore damage, seed predation or parasitism than (a) populations of the same species in their native range, or (b) populations of native plants in the introduced range [3,5–7]. This reduction in biotic pressure has direct consequences for population growth rate, and can also allow introduced species to allocate more energy to growth and reproduction, thus boosting their competitive ability and facilitating spread to further new territory [8,9].

The same filters that cause species to become separated from their enemies following introduction could also cause species to become separated from their mutualists [10]. That is, when a species is introduced to a new range, it may leave behind pollinators, seed dispersers, cleaners, defensive partners or beneficial microbes. Previous work has shown that a reduction in mutualistic interactions such as seed dispersal and pollination can lead to reduced fitness [11,12]. That is, loss of mutualisms could reduce the success of introduced species. Several authors have noted that introduced species may become separated from their mutualists [10,13–17], and this has been labelled the missed mutualist hypothesis [13]. However, the vast majority of attention on biotic interactions in invasion biology has been focused on introduced species being released from antagonistic interactions [3,18], or on the development of novel mutualisms between introduced species and co-occurring species that facilitate invasion of a new range [16,17,19,20]. A search of the ISI Web of Science (6/5/22) revealed only three papers that mentioned the missed mutualist hypothesis (containing the phrase ‘miss* mutualis*’), compared to 1084 papers containing the phrase ‘enemy release’. Clearly, there is significant bias within the existing literature on ecological invasions: we have paid a disproportionate amount of attention to the factors that promote biological invasions, with far less research effort going into studies of how losing mutualists results in the failure, or at least reduced reproductive success, of invasive taxa. However, if we want to understand the forces shaping the establishment success and evolutionary trajectory of introduced species, we need to understand both the positive and the negative selective pressures they face [10,13,19].

In this paper, we explore when species might become separated from their mutualists, then review the existing evidence for the missed mutualist hypothesis in seed dispersers, pollinators, soil microbes and endophytes, and the limited available evidence for the fitness effects of missed mutualists. We use meta-analysis to formally synthesize the available data on standardized interaction frequency for species in their native versus introduced ranges. We discuss ways introduced species can mitigate the effects of missed mutualisms through formation of new partnerships and/or through evolutionary change. Finally, we outline the practical implications of missed mutualisms, and discuss how this might guide policy and action on introduced species.

It is worth bearing in mind that data comparing interaction strength for species in native and introduced ranges can only exist for species that have successfully established in a new range. That is, our study necessarily excludes those species that failed to establish viable populations in their new range because they were not able to persist without their mutualists. Thus, this paper provides a conservative estimate of the true importance of missed mutualists.

## 2. When might species become separated from their mutualists?

There are at least three ways in which a species could become separated from a mutualistic partner species when it is introduced to a new range:

(1) *The two species may not both be introduced to the same place*. This is quite likely, as biosecurity laws in most countries limit the import of species, and have provisions that reduce the likelihood that intentionally imported species are carrying individuals or propagules of other taxa [21,22].
(2) *The mutualist species may not be able to tolerate the biotic and abiotic conditions in the new range.* This is also likely, as introduced species often establish in areas in which abiotic conditions differ from those in their home range [23] so differences in the fundamental niche space of the introduced species and their mutualists may preclude co-occurrence in the new range. Different biotic pressures in the new range may also prevent a species' establishment in a new range. For example, the interacting species might be unable to sustain a viable population in the presence of novel predators or pathogens in the new range, or they may fail to establish because they are separated from another species with which they have crucial mutualistic interactions.
(3) *The interacting partner species might fail to establish a viable population in the new range due to stochastic effects.* Again, this is relatively likely as populations are highly vulnerable to stochastic effects in the early stages of establishment in a new range [24], and only a small fraction of introductions succeed [25].

We can think of two situations in which introduced species would not lose their coevolved mutualists. First, mutualists may be successfully introduced (either deliberately or accidentally) with their interaction partner (co-introduced associations *sensu* [26], e.g. some fig trees (*Ficus* spp.) and their specialist pollinators [27,28]), or introduced to a region where a mutualist has already been introduced [16] (e.g. many plants introduced to Europe are pollinated by introduced bumblebees (*Bombus* spp.) [29]). Second, some mutualists may have a distribution that already spans the partner's native and introduced ranges (familiar associations, *sensu* [26], e.g. the hawkmoth pollinator of *Lilium formosanum* [30] and the mycorrhizal partner of the orchid *Oeceoclades maculata* [31]). However, while there are examples of both of these situations, it seems likely that these are the exceptions rather than the rule. First, most species have restricted ranges rather than cosmopolitan distributions [32]. Second, while generalist mutualists might already be established in a new range prior to a species' arrival, this is less likely to apply to more specialized interaction partners [3]. Finally, import of both partners may be restricted, as biosecurity risk assessments (e.g. in Australia and the USA [33,34]) consider risks associated with mutualisms when assessing the likelihood of invasion success. For example, when a given plant species for introduction is dependent on specialist pollinators for reproduction, it is regarded as a lower risk when introduced without its pollinators. Similarly, nitrogen-fixing plants are thought under both Australian and USA systems to have an elevated risk of becoming weedy because of their ability to form association with rhizobia bacteria to overcome edaphic abiotic limitations in the invaded range [33,34].

About half of introduced species experience reduced damage from enemies in their new ranges [2,3,6]. As most filters on the introduction of interacting species to new ranges seem equally apply to enemies as to mutualists, we might expect missing mutualists to affect a similar proportion of introduced species as enemy release. However, neither the relative extent nor the impact of enemy versus missed mutualisms has been quantitatively explored at the cross-species level.

## 3. Evidence for the missed mutualist hypothesis

Under the missed mutualist hypothesis, we predict that introduced species will: (a) interact with fewer species of mutualists in their introduced range than in their native range; and (b) interact with mutualists at a lower frequency in the introduced range than in their native range. We have focused on interactions between plants and their seed dispersers, pollinators, soil microbes and endophytes, as these are known to influence plant species fitness by impacting survival, growth and reproduction. There are many excellent studies of interactions between introduced species and other native and introduced organisms in the novel range [16]. For example, there are several cases in which the flowers of introduced species attract native pollinators and decrease the pollination success of native plants [17]. However, such studies cannot tell us whether a species experiences fewer interactions with mutualists in their introduced range than in their native range, as they are done entirely in the introduced range. Thus, our focus here is on studies that present quantitative data for interactions with mutualists in both the native and the introduced range.

### (a) Seed dispersal

We know that seed dispersal can be important in facilitating invasions and that some successful invaders are dispersed by native and introduced animals (evidence reviewed in [16]). However, surprisingly few studies have quantified seed dispersal rates or disperser diversity in both native and introduced ranges. *Acacia dealbata* has 1.4 times, and *A. longifolia* has 1.5 times as many species of seed dispersers in their native range (Australia) as in their introduced range (Portugal) [35]. There was also a decreased interaction frequency in the introduced range: *A. dealbata* has 8.6 times and *A. longifolia* has 13.9 times as many interactions per minute with seed dispersers in Australia as in Portugal [35]. Similarly, *A. dealbata, A. baileyana and A. pravissima* all show higher rates of removal (1.2, 3.8 and 1.4 times greater respectively) by invertebrate dispersers in Australia (native range) than in New Zealand (introduced range) [36]. Thus, while quantitative data are scarce, the available data are consistent with the missed mutualist hypothesis.

### (b) Pollination

The idea that introduced species are most likely to be successful if they do not rely on specialist pollinators has a long history in invasion biology [16,17,37]. There is also evidence that some (but not all) introduced populations experience pollen limitation [16]. The quantitative evidence for differences in plant–pollinator interactions between species’ native and introduced ranges is mixed, but tends to support the missed mutualist hypothesis. Emer *et al.* [38] assessed the ecological roles of species of pollinators and plants in networks where they were native compared to where they were introduced, finding that seven species had fewer interaction partners in the introduced range, five species had a greater number of partners, and four species had approximately the same number of partners. Double the flower visitation rate and almost twice as many pollinating species have been recorded for *Rhododendron ponticum* in native Iberian populations than in its introduced range in Ireland [39]. Average pollinator richness in native populations of *Nicotiana glauca* in South America is twice that of introduced populations in South Africa although there are no differences in visitation rates [40,41]. Visitation rates to *Hedysarum coronarium* are more than five times greater in its native range, where pollinator diversity is about 30% higher [42]. Visitation rates to flowers of introduced populations of *Solanum elaeagnifolium* are 1.6 times higher on average than to native populations, however, no pollinators were observed for many introduced populations, and average pollinator diversity was more than five times higher in the native range [43]. *Datura stramonium* populations in the native range have 25% fewer pollinator species, although the average visitation rate is three times higher [44]. Finally, in an example of highly specialized, co-evolved mutualist pollinator loss, the diversity of fig wasps pollinating *Ficus rubiginosa* has dropped from five in its native Australia to one in non-native populations in the northern hemisphere [27].

### (c) Soil microbes

Evidence from legume–rhizobacteria interactions tends to support the predictions of the missed mutualist hypothesis. Experimental studies in clover species (*Trifolium* spp.) have shown that colonization by *Rhizobia* spp. was generally higher in soils of the native range compared to the introduced range [45]. These legume species are likely to lose mutualists in the event of being translocated from their native range. The evidence for this from other legumes (*Acacia* spp., *Mimosa* spp.) indicates that most species are promiscuous in having broader acceptability of rhizobacterial mutualists [46–48]. However, even within these genera some species have high fidelity to specific rhizobacteria (e.g. *M. hamata* [46]), which could limit their success outside of their native range. Some introduced *Acacia* spp. rely on the co-introduction of their symbionts, rather than on novel mutualisms in their introduced ranges [49].

The obligate interactions of plants with mycorrhizae present another opportunity for the loss of key mutualists in the translocations of such plants. For example, different orchids exhibit obligate and facultative mycorrhizal associations and these relationships, at least in part, explain their distribution, abundance and invasiveness [50].

### (d) Endophytes

In the case of endophyte–plant interactions, evidence for the effect of losing specialist mutualisms in introduced ranges is strongest for grasses. For example, the loss of vertically transmitted mutualists in *Poa*–*Neophytidium* interactions can be detrimental to successful establishment of grasses in novel ranges [15]. Evidence for specialist endophytic mutualisms in eudicots is less definitive than for grasses, but there is a reduction in endophyte diversity in *Theobroma* spp. from the native range (South America) to introduced cultivated contexts [51,52]. The relatively higher susceptibility to disease of cultivated plants indicates a possible cost to the loss of endophyte diversity [53], in line with the predictions of the missed mutualist hypothesis.

### (e) Quantitative support for the missed mutualist hypothesis

**Figure 1.**
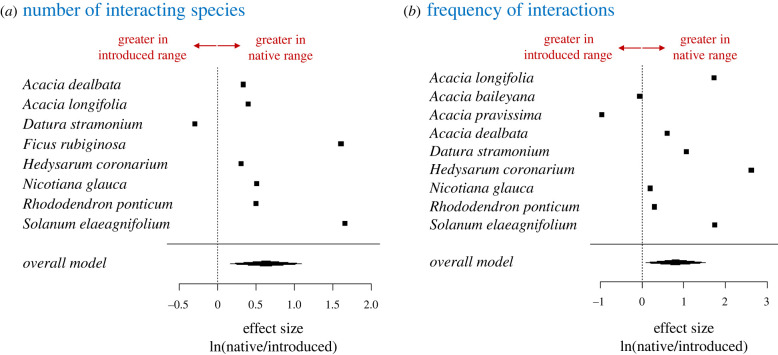
Forest plots showing the effect sizes for species for which (*a*) the number of interacting mutualist species or (*b*) the frequency of mutualist interactions were measured using consistent methods in both the species’ native and introduced ranges within the same study. The effect sizes are log response ratios [ln(native/introduced)]. Values greater than zero indicate more interacting species or more frequent interactions in the native range. The diamonds represent the overall estimated effect size.

We provided the first cross-species, quantitative test of the predictions of the missed mutualist hypothesis using meta-analysis of data from studies in which a species' interactions with mutualists had been quantified in the same way in both their native and introduced ranges (see electronic supplementary material, appendix 1 for methods and electronic supplementary material, appendix 2 for raw data). Data were compiled using systematic literature searches for pollination and seed dispersal, the only interaction types for which field-based quantitative data were available. Unfortunately, relatively few such studies are available at present. Despite this, analysis showed that species interact with 1.9 times fewer species in their introduced range than in their native range (overall effect size [log response ratio] = 0.63, *n* = 8 species, *p* = 0.008; figure 1*a*). Plants also have significantly lower interaction frequencies in their introduced ranges (overall effect size = 0.81, *n* = 9 species, *p* = 0.026; figure 1*b*). That is, plants have 2.3 times more interactions with mutualists per unit time in their native range than in their introduced range. These findings are in stark contrast to previous suggestions that introduced species readily acquire new mutualists in their new range [16].

The available data are too sparse to do a meaningful comparison of the proportion of species experiencing enemy release versus the proportion of species experiencing missing mutualisms. However, with seven out of the eight species for which quantitative data are available interacting with more species in their native range than in their introduced range, and seven out of nine species having a higher frequency of interactions in their native range than in their introduced range (figure 1), it seems possible that missed mutualisms may actually be as common, or even more common than enemy release. There will also be many cases in which both enemy release and missed mutualisms affect fitness, and in these cases, quantifying the relative magnitude of the effects is an important direction for the future. On balance, given the success of introduced species, it seems likely that the positive effects of shedding biotic interactions might outweigh the negative. Another interesting direction for future research is to quantify the trajectory of missed mutualists through the invasion process. One possibility is that the effect is strongest soon after introduction when the number of individuals of the introduced species is very low [54].

The meta-analysis provides relatively strong support for the two predictions of the missed mutualist hypothesis (figure 1). However, data comparing interaction strength for species in native and introduced ranges can only exist for species that have successfully established in a new range, and so our analysis only captures some of the effect of missed mutualisms.

## 4. Evidence for fitness effects of missed mutualists

**Figure 2.**
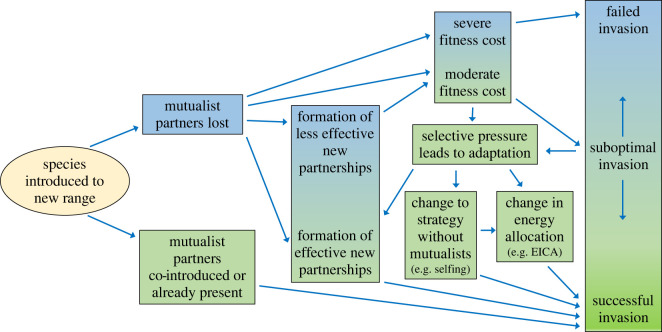
The effects of missed mutualisms, and pathways through which species can reduce negative effects. Boxes with a gradient of colour represent effects that can occur at a range of strengths. For simplicity, this diagram only shows the effects of missed mutualists. However, abiotic conditions and enemy release could counterbalance or exacerbate negative effects on introduced populations. EICA, evolution of increased competitive ability.

The reduced number of interacting species, and particularly the lower frequency of interactions experienced by species in their introduced range could have immediate demographic effects (figure 2). For instance, missed interactions with symbiotic soil microbes could limit resource capture and thus decrease growth and reproduction [55]. Diminished pollination could limit seed set [56], and reduced seed dispersal could result in offspring remaining near their maternal plants and thus experiencing distance and density-dependent mortality caused by higher competition, predation or pathogen attack [57].

There is some evidence for introduced populations suffering decreased fitness as a result of a lack of microbial mutualists. For example, the absence of *Ensifer medicae,* a rhizobial mutualist, was associated with a dramatic decline in shoot mass of *Medicago polymorpha* [58]*.* Similarly, a study of the effect of soil microbes on *Pinus contorta* (a highly invasive member of the genus *Pinus*, which is an ectomycorrhizal clade) found that seedlings grown in sterile soil gained twice as much biomass when inoculated with soil from the native range than when inoculated with soil from the introduced range [59]. However, not all the evidence is in line with the missed mutualist hypothesis. A study of introduced *Pinus ponderosa* in Argentina found evidence for decreasing ectomycorrhizal colonization of roots with increasing distances from plantations, but no significant effect on seedling growth or survival [60].

Having fewer pollinators may not always result in decreased fitness in introduced populations, as pollination efficacy can vary. For example, pollinators in the invasive range may be more effective at transferring pollen [61]. The invasive, pollinator-dependent, annual plant Himalayan balsam (*Impatiens glandulifera*) is a potentially interesting case study in this regard. This species has a very high rate of nectar production such that it attracts pollinators away from native plants, reducing their seed set [62]. A separate study in Britain showed that the mean number of seeds per fruit and the mean number of fruit per plant can vary by an order of magnitude depending on the population [63]. Despite attracting many pollinators in Europe, *I. glandulifera* populations are clearly not always performing at their optimum reproductive output. There are of course other possible explanations for the low seed production, but if it is due to the recruitment of fewer, sub-optimum pollinators, this could be a significant potential barrier to establishment, in line with the missed mutualist hypothesis.

The fitness effects of missed seed dispersers or pollinators can be difficult to disentangle from other processes. For instance, although introduced populations often experience pollen limitation (invasive species experience significantly less pollen limitation than do related native species or non-invasive introduced species [64]), invasive plants can maintain high reproductive success through a high number of flowers per plant [64]. Similarly, introduced plant populations tend to have greater seed production than native populations [65], but these data aggregate the effects of enemy release (reduced damage from herbivores, seed predators and pathogens and potential for decreased investment in defences, leaving more energy for seed production), and missed mutualists (less pollination and thus potentially decreased seed set).

As with evidence for the abundance or diversity of mutualists in the new range, the observable fitness effects of missed mutualisms do not capture the most extreme cases. Species with obligate mutualisms that arrive in a new range without their mutualists will simply fail to establish [60]. A worthwhile direction for future research is to try to estimate what proportion of invasions fail because of a lack of mutualists. An intriguing possibility is that this proportion may have decreased through the last few hundred years, as the number of introduced species present worldwide increased [66].

## 5. Evolutionary implications of missed mutualists

We know relatively little about the evolutionary responses of plants to the loss of mutualists in their new ranges. By contrast, the evolutionary implications of enemy release have been explored through the evolution of increased competitive ability (EICA) hypothesis. EICA predicts that reduced herbivore pressure allows plants to decrease resource allocation to defence and increase resource allocation to growth, resulting in increased competitive ability relative to native plants [8]. Mutualisms also require an investment in resources, e.g. in scent components and nectar for attracting and rewarding pollinators; or in maintaining mutualistic microbes within plant tissues. Thus there may also be evolutionary implications for the missed mutualist hypothesis. However, in missing mutualists, the reduced costs of investment in resources may be balanced by a loss of fitness due to the absence of the mutualist. For example, pollen limitation is frequently observed in introduced species in their new range [64,67,68]. Likewise, loss of seed dispersers [12] and pollinators [69] has been implicated in the declines of several species (consistent with the idea that there will also be selective pressures associated with missed mutualists). Populations that successfully establish in a new range but suffer missed mutualisms seem likely to either evolve so that they can survive without their mutualists or evolve to facilitate relationships with alternative mutualists [26,58] (figure 2).

### (a) Evolving to survive without mutualists

Introduced species may shift from biotically mediated reproduction to abiotically- or self-mediated reproduction. Evidence for this expectation in seed dispersal generally appears lacking. By contrast, evolutionary shifts in plant mating systems to promote self-fertilization following their shifts into new ranges are frequently observed [70], such as instances of shifts from biotic to abiotic mediated pollination (e.g. where self-pollination is facilitated by rainfall) [71,72]. For instance, wind pollination tends to increase at range edges where population density is low [73]. Similarly, plants at the edges of their range or in expanding range fronts can shift mating systems toward self-fertilization [74] or clonal reproduction [75]. *Nicotiana glauca*, for example*,* is a hummingbird pollinated in its native range, and introduced populations in regions with no specialized bird pollinators have switched to selfing [40,41]. Evolutionary modelling indicates that self-fertilization should be selected over outcrossing as long as the offspring of selfers are at least half as fit as the offspring of outcrossers [76,77]. Thus, solutions to the loss of pollination (and/or dispersal) interactions may rapidly evolve and fix in introduced populations rapidly given that being unsuccessful in reproduction has an ultimate fitness cost and threatens the population with extinction [78].

The loss of mutualists could result in selection for changes in energy allocation. For example, *Acacia* elaiosomes (lipid-rich seed appendages which encourage dispersal by ants) are of less importance in their introduced range than in the native range despite ants remaining the dispersal vector [35], and over time introduced *Acacia* populations may be reducing their investment in elaiosomes [79,80]. The redirection of this investment to greater seed numbers or increased seed provisioning could potentially increase the colonization success or competitiveness of introduced populations. That is, release from enemies is not the only mechanism for the EICA.

### (b) Evolving to facilitate interactions with new partners

It is common for species to undergo substantial and rapid phenotypic and genetic changes after being introduced to new ranges [81–85]. The missed mutualist hypothesis provides insights into the type of selective pressures experienced by species in the early stages of biological invasions [58]. As the mutualisms involved with pollination, seed dispersal and nitrogen fixation can be both highly specialized and critical to a plants’ fitness, selection on traits associated with these mutualisms could be extremely strong. This selection might lead to substantial morphological or genetic changes over short periods and may contribute to the development of reproductive isolation between native and introduced populations (such as that recorded by [86]).

We used the Pollinators of the Apocynaceae Database [87] to quantify how often species change their pollination system when they are introduced to a new range (electronic supplementary material, figure S1). In over a quarter of the examples (11/42), species switched pollination systems. Four species switched from being pollinated by a limited set of related species in the native range to being more ecologically generalized and using diverse pollinators in the introduced range. Conversely, three species that were generalists in their native range have become more functionally specialized after introduction because they left behind some types of mutualists. The mixed pattern of species that are conservative and those that are more flexible in their pollination systems in electronic supplementary material, figure S1 is not confined to Apocynaceae; the literature abounds with examples of species switching [88–92] or maintaining their pollinator type in a new range [40,41]. The switches in pollinators shown here could be either plasticity or evolution. However, interacting with different pollinators seems likely to induce evolution over the longer term.

Pollinators can impose strong selection on floral traits such as flower size, colour, odour and nectar characteristics, as well as phenological traits such as the timing of flowering [93,94]. Thus, switches to new types of pollinators in an introduced range could result in the evolution of changes in reproductive characters that enhance such interactions. This idea is supported by observed changes in the size [95], width [96], floral tube shape and length [93] and nectar chemistry [97] of flowers of introduced populations in response to the pollinating species in their new range. A particularly nice example comes from *Digitalis purpurea*, which is pollinated by bumblebees in its native range, and by both bees and hummingbirds in the neotropics (where it was introduced in the approx. 1850s). Consistent with this change in pollinator, *D. purpurea* populations in the neotropics have evolved longer proximal corolla tubes [98].

## 6. Practical implications of missed mutualists

About 60% of species fail to transition from introduction to establishment in a new range [99]. Some of these failed invasions might be explained by separation from obligate mutualists [13,26]. For example, the invasion of Pinaceae in Argentina is hindered by the absence of ectomycorrhizal fungi [18]. Plants that are obligately reliant on their mutualist pollinators may be restricted from establishing successful introduced populations: species with lower or no selfing ability are less likely to become naturalized or invasive outside their native range than their sympatric selfing congeners [100]. Further evidence to support the idea that the loss of mutualists could impose a strong filter on colonization of new areas comes from taxa that have not become established as introduced species despite significant opportunities for them to do so. For example, anemonefishes (*Amphiprion* spp. and *Premnas biaculeatus*) have an obligate association with sea anemones (Actinaria) that are restricted to the Indo-Pacific region [101]. The success of the movie *Finding Nemo* led to anemonefish becoming more common in aquaria. Despite this, populations have not established around the Caribbean or Mediterranean Seas, where unwanted fish are frequently released into the ocean [102,103].

Many introduced species experience a lag phase—a gap between the initial establishment of the population and the onset of rapid population increase [104]. The loss of mutualists is one of several factors that could contribute to these lag phases [13,19,26,59]. For instance, a lag phase could result from the gap between the arrival of an introduced species and the arrival of mutualists from its home range. Alternatively, a lag phase might result from the time taken for the introduced species to undergo rapid evolution of traits to facilitate successful interactions with species that are already present in the novel range.

The overall fitness implications of establishing in a new range will depend on the balance of the positive effects of enemy release and the negative effects of missed mutualists, in addition to the species' ability to tolerate the abiotic conditions [10,13,59]. The long-term success of biological invasions will also depend on how fast introduced species accumulate new enemies or mutualists [13,16]. If new enemies accumulate faster than new mutualists, then the success of invasions may be limited even under circumstances where new mutualisms can evolve.

### (a) Managing risks of introduced species in the context of missed mutualists

The enemy release hypothesis has provided a useful conceptual framework for developing management tools for introduced species. For example, where release from specialist antagonists is an important factor in the success of an invader, biological control approaches using specialist natural enemies in the invaded range can have value [3,81,105]. The missed mutualist hypothesis could potentially offer similar guidance on anticipating and managing risks posed by introduced species that are separated from their mutualists.

Introduced species in obligate or specialized mutualistic interactions in their native range are less likely to become invasive than those that are not reliant on such specialized interactions [16–18]. Prospective biosecurity risk assessment tools (e.g. Australian Weed Risk Assessment [34]) therefore are more tolerant of introductions of invaders that have been separated from their mutualists (e.g. specialist pollinators). To enhance such risk assessments, it would be valuable to consider whether there are analogues to the lost mutualists in the invaded range of the introduced species, and the likelihood for acquisition of new mutualists or the subsequent arrival of old mutualists. For instance, the spread of introduced Pinaceae can be limited by a lack of associated ectomycorrhizal fungi [18]. However, mycorrhizal inoculum is used globally, almost totally unchecked, to support the establishment of successful plantations of Pinaceae [106], which has the potential to disrupt current range expansion barriers for some pines [18,107,108]. The impacts of agricultural or forestry practices which could overcome barriers to invasion associated with missed mutualists should be explored. Another biosecurity risk assessment strategy could identify those species for which missing mutualists are less likely to encumber successful establishment. For instance, examining the plasticity in breeding systems and the relative importance/frequency of self- versus cross-pollination in the native range may shed light on the likelihood for plants to become invasive despite the absence of their mutualists in the new range [109,110]. Finally, in cases where invaders and their mutualists were co-introduced, we could use understanding of the importance of mutualists in introduced populations to identify introduced species that might become locally extinct if their mutualist were removed.

For introduced species that acquire new mutualists in the introduced range and consequently become invasive, management tactics may need to be developed that disrupt novel interactions. For example, managing populations of introduced legumes that are promiscuous (i.e. do not need specialized rhizobial mutualists and can acquire and use local rhizobacteria in the invaded range), may require disruption of the formation efficiency of such symbiotic associations (e.g. periodic manipulation of soil pH [111]). As is the case for management applications based on the enemy release hypothesis, the off-target/undesirable effects of any such management manipulations need to be carefully considered [112].

## 7. Conclusion

The effect of missed mutualisms on introduced species can vary from a complete failure of invasion to a temporary setback that can be overcome through formation of new mutualisms, evolution of a strategy that does not require mutualists, or a change in energy allocation (figure 2). We hope this paper will stimulate invasion biologists to further develop ideas about the implications of the full suite of biotic interactions in determining the success or failure of species introductions, and to explore the ecological and evolutionary consequences of disrupted mutualisms in introduced species. Given the rising economic and environmental costs of invasive species worldwide [113], new avenues for improved mitigation and prevention of invasion are valuable. Much in the way that the enemy release hypothesis has been useful to biocontrol and management of introduced species, we hope that the missed mutualist hypothesis will be applied to help manage species introductions in the future.

## Data Availability

The raw dataset is included in electronic supplementary material, appendix 2 [114].

## References

[1] Liu H, Stiling P. 2006 Testing the enemy release hypothesis: a review and meta-analysis. Biol. Invasions **8**, 1535-1545. (10.1007/s10530-005-5845-y)

[2] Colautti RI, Ricciardi A, Grigorovich IA, MacIsaac HJ. 2004 Is invasion success explained by the enemy release hypothesis? Ecol. Lett. **7**, 721-733. (10.1111/j.1461-0248.2004.00616.x)

[3] Keane RM, Crawley MJ. 2002 Exotic plant invasions and the enemy release hypothesis. Trends Ecol. Evol. **17**, 164-170. (10.1016/S0169-5347(02)02499-0)

[4] Leishman MR, Cooke J, Richardson DM. 2014 Evidence for shifts to faster growth strategies in the new ranges of invasive alien plants. J. Ecol. **102**, 1451-1461. (10.1111/1365-2745.12318) 25558090 PMC4277856

[5] Fenner M, Lee WG. 2001 Lack of pre-dispersal seed predators in introduced Asteraceae in New Zealand. N. Z. J. Ecol. **25**, 95-99.

[6] Roy HE, Lawson Handley LJ, Schönrogge K, Poland RL, Purse BV. 2011 Can the enemy release hypothesis explain the success of invasive alien predators and parasitoids? BioControl **56**, 451-468. (10.1007/s10526-011-9349-7)

[7] Torchin ME, Mitchell CE. 2004 Parasites, pathogens, and invasions by plants and animals. Front. Ecol. Environ. **2**, 183-190. (10.1890/1540-9295(2004)002[0183:PPAIBP]2.0.CO;2)

[8] Blossey B, Nötzold R. 1995 Evolution of increased competitive ability in invasive nonindigenous plants: a hypothesis. J. Ecol. **83**, 887-889. (10.2307/2261425)

[9] Callaway RM, Ridenour WM. 2004 Novel weapons: invasive success and the evolution of increased competitive ability. Front. Ecol. Environ. **2**, 436-443. (10.1890/1540-9295(2004)002[0436:NWISAT]2.0.CO;2)

[10] Mitchell CE et al. 2006 Biotic interactions and plant invasions. Ecol. Lett. **9**, 726-740. (10.1111/j.1461-0248.2006.00908.x) 16706916

[11] Bodbyl Roels SA, Kelly JK. 2011 Rapid evolution caused by pollinator loss in *Mimulus guttatus*. Evolution **65**, 2541-2552. (10.1111/j.1558-5646.2011.01326.x) 21884055 PMC5958604

[12] Egerer MH, Fricke EC, Rogers HS. 2018 Seed dispersal as an ecosystem service: frugivore loss leads to decline of a socially valued plant, *Capsicum frutescens*. Ecol. Appl. **28**, 655-667. (10.1002/eap.1667) 29271019 PMC5947168

[13] Alpert P. 2006 The advantages and disadvantages of being introduced. Biol. Invasions **8**, 1523-1534. (10.1007/s10530-005-5844-z)

[14] Catford JA, Jansson R, Nilsson C. 2009 Reducing redundancy in invasion ecology by integrating hypotheses into a single theoretical framework. Divers. Distrib. **15**, 22-40. (10.1111/j.1472-4642.2008.00521.x)

[15] Evans H. 2008 The endophyte-enemy release hypothesis: implications for classical biological control and plant invasions. In Proc. of the XII Int. Symp. on Biological Control of Weeds, vol. 22, pp. 20-26. (10.1079/9781845935061.0020).

[16] Richardson DM, Allsopp N, D'Antonio CM, Milton SJ, Rejmánek M. 2000 Plant invasions—the role of mutualisms. Biol. Rev. **75**, 65-93. (10.1017/S0006323199005435) 10740893

[17] Traveset A, Richardson DM. 2014 Mutualistic interactions and biological invasions. Ann. Rev. Ecol. Evol. Syst. **45**, 89-113. (10.1146/annurev-ecolsys-120213-091857)

[18] Nuñez MA, Horton TR, Simberloff D. 2009 Lack of belowground mutualisms hinders Pinaceae invasions. Ecology **90**, 2352-2359. (10.1890/08-2139.1) 19769113

[19] Traveset A, Richardson DM. 2020 Plant invasions: the role of biotic interactions. Wallingford, UK: CABI.

[20] Vizentin-Bugoni J et al. 2021 Ecological correlates of species' roles in highly invaded seed dispersal networks. Proc. Natl Acad. Sci. USA **118**, e2009532118. (10.1073/pnas.2009532118) 33431649 PMC7848469

[21] Anderson K, McRae C, Wilson D. 2012 The economics of quarantine and the SPS agreement. Adelaide, Australia: University of Adelaide Press.

[22] Stewart TP, Johanson DS. 2003 A nexus of trade and the environment: the relationship between the Cartagena Protocol on Biosafety and the SPS Agreement of the World Trade Organization. Colo. J. Int. Env. Law & Policy **14**, 1.

[23] Gallagher RV, Beaumont LJ, Hughes L, Leishman MR. 2010 Evidence for climatic niche and biome shifts between native and novel ranges in plant species introduced to Australia. J. Ecol. **98**, 790-799. (10.1111/j.1365-2745.2010.01677.x)

[24] Simberloff D. 2009 The role of propagule pressure in biological invasions. Ann. Rev. Ecol. Evol. Syst. **40**, 81-102. (10.1146/annurev.ecolsys.110308.120304)

[25] Williamson M, Fitter A. 1996 The varying success of invaders. Ecology **77**, 1661-1666. (10.2307/2265769)

[26] Dickie IA et al. 2017 The emerging science of linked plant–fungal invasions. New Phytol. **215**, 1314-1332. (10.1111/nph.14657) 28649741

[27] Morgan E, Sutton TL, Darwell CT, Cook JM. 2018 Restructuring of a mutualism following introduction of Australian fig trees and pollinating wasps to Europe and the USA. Biol. Invasions **20**, 3037-3045. (10.1007/s10530-018-1775-3)

[28] Vianna-Filho MD, Alves RJV, Peng Y-Q, Pereira RAS. 2017 Naturalization of the Bodhi fig tree (*Ficus religiosa* L.—Moraceae) in Brazil. Bioscience J. **33**, 177-182. (10.14393/BJ-v33n1a2017-34177)

[29] Goulson D. 2003 Effects of introduced bees on native ecosystems. Ann. Rev. Ecol. Evol. Syst. **34**, 1-26. (10.1146/annurev.ecolsys.34.011802.132355)

[30] Rodger JG, van Kleunen M, Johnson SD. 2010 Does specialized pollination impede plant invasions? Int. J. Plant. Sci. **171**, 382-391. (10.1086/651226)

[31] Riverón-Giró FB, Raventós J, Damon A, García-González A, Mújica E. 2019 Spatio-temporal dynamics of the invasive orchid *Oeceoclades maculata* (Orchidaceae), in four different habitats in southeast Chiapas, Mexico. Biol. Invasions **21**, 1905-1919. (10.1007/s10530-019-01945-7)

[32] Gaston KJ. 1996 Species-range-size distributions: patterns, mechanisms and implications. Trends Ecol. Evol. **11**, 197-201. (10.1016/0169-5347(96)10027-6) 21237808

[33] Koop AL, Fowler L, Newton LP, Caton BP. 2012 Development and validation of a weed screening tool for the United States. Biol. Invasions **14**, 273-294. (10.1007/s10530-011-0061-4)

[34] Pheloung P, Williams P, Halloy S. 1999 A weed risk assessment model for use as a biosecurity tool evaluating plant introductions. J. Environ. Manage. **57**, 239-251. (10.1006/jema.1999.0297)

[35] Montesinos D, Correia M, Castro S, French K, Rodríguez-Echeverría S. 2018 Diminishing importance of elaiosomes for *Acacia* seed removal in non-native ranges. Evol. Ecol. **32**, 601-621. (10.1007/s10682-018-9959-y)

[36] Wandrag E, Sheppard A, Duncan R, Hulme P. 2013 Mutualism vs. antagonism in introduced and native ranges: can seed dispersal and predation determine *Acacia* invasion success? Perspect. Plant. Ecol. **15**, 171-179. (10.1016/j.ppees.2013.03.002)

[37] Baker HG. 1965 Characteristics and modes of origin of weeds. In The genetics of colonizing species (eds HG Baker, GL Stebbins), pp. 147-168. New York, NY: Academic Press.

[38] Emer C, Memmott J, Vaughan IP, Montoya D, Tylianakis JM. 2016 Species roles in plant–pollinator communities are conserved across native and alien ranges. Divers. Distrib. **22**, 841-852. (10.1111/ddi.12458)

[39] Stout JC, Parnell JA, Arroyo J, Crowe TP. 2006 Pollination ecology and seed production of *Rhododendron ponticum* in native and exotic habitats. Biodivers. Conserv. **15**, 755-777. (10.1007/s10531-004-1065-5)

[40] Issaly EA, Sérsic AN, Pauw A, Cocucci AA, Traveset A, Benitez-Vieyra SM, Paiaro V. 2020 Reproductive ecology of the bird-pollinated *Nicotiana glauca* across native and introduced ranges with contrasting pollination environments. Biol. Invasions. **22**, 485-498. (10.1007/s10530-019-02104-8)

[41] Ollerton J et al. 2012 Pollination ecology of the invasive tree tobacco *Nicotiana glauca*: comparisons across native and non-native ranges. J. Pollination Ecol. **9**, 85-95. (10.26786/1920-7603(2012)12)

[42] Montero-Castaño A, Vilà M, Ortiz-Sánchez FJ. 2014 Pollination ecology of a plant in its native and introduced areas. Acta Oecol. **56**, 1-9. (10.1016/j.actao.2014.01.001)

[43] Petanidou T et al. 2018 Pollination and reproduction of an invasive plant inside and outside its ancestral range. Acta Oecol. **89**, 11-20. (10.1016/j.actao.2018.03.008)

[44] Jiménez-Lobato V, Martínez-Borda E, Núñez-Farfán J, Valverde P, Cruz L, López-Velázquez A, Santos-Gally R, Arroyo J. 2018 Changes in floral biology and inbreeding depression in native and invaded regions of *Datura stramonium*. Plant Biol. **20**, 214-223. (10.1111/plb.12658) 29106048

[45] Shelby N, Duncan RP, Van Der Putten WH, McGinn KJ, Weser C, Hulme PE. 2016 Plant mutualisms with rhizosphere microbiota in introduced versus native ranges. J. Ecol. **104**, 1259-1270. (10.1111/1365-2745.12609)

[46] Gehlot HS et al. 2013 An invasive *Mimosa* in India does not adopt the symbionts of its native relatives. Ann. Bot. **112**, 179-196. (10.1093/aob/mct112) 23712450 PMC3690997

[47] Keet J-H, Ellis AG, Hui C, Le Roux JJ. 2017 Legume–rhizobium symbiotic promiscuity and effectiveness do not affect plant invasiveness. Ann. Bot. **119**, 1319-1331. (10.1093/aob/mcx028) 28369229 PMC5604570

[48] Klock MM, Barrett LG, Thrall PH, Harms KE. 2016 Differential plant invasiveness is not always driven by host promiscuity with bacterial symbionts. AoB Plants **8**, plw060. (10.1093/aobpla/plw060) 27535176 PMC5018393

[49] Rodríguez-Echeverría S, Fajardo S, Ruiz-Díez B, Fernández-Pascual M. 2012 Differential effectiveness of novel and old legume–rhizobia mutualisms: implications for invasion by exotic legumes. Oecologia **170**, 253-261. (10.1007/s00442-012-2299-7) 22419481

[50] Ackerman J. 2007 Invasive orchids: weeds we hate to love? Lankesteriana **7**, 19-21. (10.15517/lank.v7i1-2.18386)

[51] Crozier J, Thomas S, Aime M, Evans H, Holmes K. 2006 Molecular characterization of fungal endophytic morphospecies isolated from stems and pods of *Theobroma cacao*. Plant. Pathol. **55**, 783-791. (10.1111/j.1365-3059.2006.01446.x)

[52] Evans HC, Holmes KA, Thomas SE. 2003 Endophytes and mycoparasites associated with an indigenous forest tree, *Theobroma gileri*, in Ecuador and a preliminary assessment of their potential as biocontrol agents of cocoa diseases. Mycol. Prog. **2**, 149-160. (10.1007/s11557-006-0053-4)

[53] Hardoim PR, Van Overbeek LS, Berg G, Pirttilä AM, Compant S, Campisano A, Döring M, Sessitsch A. 2015 The hidden world within plants: ecological and evolutionary considerations for defining functioning of microbial endophytes. Microbiol. Mol. Biol. R. **79**, 293-320. (10.1128/MMBR.00050-14) PMC4488371 26136581

[54] Benadi G, Pauw A. 2018 Frequency dependence of pollinator visitation rates suggests that pollination niches can allow plant species coexistence. J. Ecol. **106**, 1892-1901. (10.1111/1365-2745.13025)

[55] Van Der Heijden MGA, Bardgett RD, Van Straalen NM. 2008 The unseen majority: soil microbes as drivers of plant diversity and productivity in terrestrial ecosystems. Ecol. Lett. **11**, 296-310. (10.1111/j.1461-0248.2007.01139.x) 18047587

[56] Knight TM et al. 2005 Pollen limitation of plant reproduction: pattern and process. Ann. Rev. Ecol. Evol. Syst. **36**, 467-497. (10.1146/annurev.ecolsys.36.102403.115320)

[57] Hansen DM, Kaiser CN, Müller CB. 2008 Seed dispersal and establishment of endangered plants on oceanic islands: the Janzen–Connell model, and the use of ecological analogues. PLoS ONE **3**, e2111. (10.1371/journal.pone.0002111) 18461169 PMC2358974

[58] Lopez ZC, Friesen ML, Von Wettberg E, New L, Porter S. 2021 Microbial mutualist distribution limits spread of the invasive legume *Medicago polymorpha*. Biol. Invasions **23**, 843-856. (10.1007/s10530-020-02404-4)

[59] Nuske SJ et al. 2021 Soil biotic and abiotic effects on seedling growth exhibit context dependent interactions: evidence from a multi-country experiment on *Pinus contorta* invasion. New Phytol. **232**, 303-317. (10.1111/nph.17449) 33966267

[60] Moyano J, Chiuffo MC, Policelli N, Nuñez MA, Rodriguez Cabal MA. 2019 The interplay between propagule pressure, seed predation and ectomycorrhizal fungi in plant invasion. NeoBiota **42**, 45-58. (10.3897/neobiota.42.30978)

[61] Moroń D, Marjańska E, Skórka P, Lenda M, Woyciechowski M. 2021 Invader–pollinator paradox: invasive goldenrods benefit from large size pollinators. Divers. Distrib. **27**, 632-641. (10.1111/ddi.13221)

[62] Chittka L, Schürkens S. 2001 Successful invasion of a floral market. Nature **411**, 653. (10.1038/35079676) 11395755

[63] Willis S, Hulme P. 2004 Environmental severity and variation in the reproductive traits of *Impatiens glandulifera*. Funct. Ecol. **18**, 887-898. (10.1111/j.0269-8463.2004.00907.x)

[64] Burns JH et al. 2019 Plant traits moderate pollen limitation of introduced and native plants: a phylogenetic meta-analysis of global scale. New Phytol. **223**, 2063-2075. (10.1111/nph.15935) 31116447

[65] Mason R, Cooke J, Moles AT, Leishman MR. 2008 Reproductive output of invasive versus native plants. Global. Ecol. Biogeogr. **17**, 633-640. (10.1111/j.1466-8238.2008.00402.x)

[66] Seebens H et al. 2017 No saturation in the accumulation of alien species worldwide. Nat. Commun. **8**, 14435. (10.1038/ncomms14435) 28198420 PMC5316856

[67] Parker IM. 1997 Pollinator limitation of *Cytisus scoparius* (Scotch broom), an invasive exotic shrub. Ecology **78**, 1457-1470. (10.1890/0012-9658(1997)078[1457:PLOCSS]2.0.CO;2)

[68] Zhang L-J, Lou A-R. 2015 Pollen limitation in invasive populations of *Solanum rostratum* and its relationship to population size. J. Plant. Ecol. **8**, 154-158. (10.1093/jpe/rtv013)

[69] Aslan CE, Liang CT, Shiels AB, Haines W. 2018 Absence of native flower visitors for the endangered Hawaiian mint *Stenogyne angustifolia*: impending ecological extinction? Glob. Ecol. Conserv. **16**, e00468. (10.1016/j.gecco.2018.e00468)

[70] Barrett SCH, Colautti RI, Eckert CG. 2008 Plant reproductive systems and evolution during biological invasion. Mol. Ecol. **17**, 373-383. (10.1111/j.1365-294X.2007.03503.x) 17868309

[71] Aguiar JM, Pansarin LM, Ackerman JD, Pansarin ER. 2012 Biotic versus abiotic pollination in *Oeceoclades maculata* (Lindl.) Lindl. (Orchidaceae). Plant. Spec. Biol. **27**, 86-95. (10.1111/j.1442-1984.2011.00330.x)

[72] González-Díaz N, Ackerman J. 1988 Pollination, fruit set, and seed production in the orchid, *Oeceoclades maculata*. Lindleyana **3**, 150-155.

[73] Friedman J, Barrett SC. 2009 Wind of change: new insights on the ecology and evolution of pollination and mating in wind-pollinated plants. Ann. Bot. **103**, 1515-1527. (10.1093/aob/mcp035) 19218583 PMC2701749

[74] Herlihy CR, Eckert CG. 2005 Evolution of self-fertilization at geographical range margins? A comparison of demographic, floral, and mating system variables in central vs. peripheral populations of *Aquilegia canadensis* (Ranunculaceae). Am. J. Bot. **92**, 744-751. (10.3732/ajb.92.4.744) 21652454

[75] Eckert CG, Barrett SC. 1993 Clonal reproduction and patterns of genotypic diversity in *Decodon verticillatus* (Lythraceae). Am. J. Bot. **80**, 1175-1182. (10.1002/j.1537-2197.1993.tb15350.x)

[76] Lande R, Schemske DW. 1985 The evolution of self-fertilization and inbreeding depression in plants. I. Genetic models. Evolution **39**, 24-40. (10.1111/j.1558-5646.1985.tb04077.x) 28563655

[77] Lloyd DG. 1992 Self-and cross-fertilization in plants. II. The selection of self-fertilization. Int. J. Plant. Sci. **153**, 370-380. (10.1086/297041)

[78] Pannell JR. 2006 Effects of colonization and metapopulation dynamics on the evolution of plant sexual systems. In Ecology and evolution of flowers (eds LD Harder, SC Barrett), pp. 223-238. Oxford, UK: Oxford University Press.

[79] Correia M, Montesinos D, French K, Rodríguez-Echeverría S. 2016 Evidence for enemy release and increased seed production and size for two invasive Australian acacias. J. Ecol. **104**, 1391-1399. (10.1111/1365-2745.12612)

[80] Harris CJ, Manea A, Moles AT, Murray BR, Leishman MR. 2017 Differences in life-cycle stage components between native and introduced ranges of five woody Fabaceae species. Austral. Ecol. **42**, 404-413. (10.1111/aec.12456)

[81] Blumenthal DM, Hufbauer RA. 2007 Increased plant size in exotic populations: a common-garden test with 14 invasive species. Ecology **88**, 2758-2765. (10.1890/06-2115.1) 18051644

[82] Bossdorf O, Auge H, Lafuma L, Rogers WE, Siemann E, Prati D. 2005 Phenotypic and genetic differentiation between native and introduced plant populations. Oecologia **144**, 1-11. (10.1007/s00442-005-0070-z) 15891837

[83] Buswell JM, Moles AT, Hartley S. 2011 Is rapid evolution common in introduced plant species? J. Ecol. **99**, 214-224. (10.1111/j.1365-2745.2010.01759.x)

[84] Dalrymple RL, Buswell JM, Moles AT. 2015 Asexual plants change just as often and just as fast as do sexual plants when introduced to a new range. Oikos **124**, 196-205. (10.1111/oik.01582)

[85] Felker-Quinn E, Schweitzer JA, Bailey JK. 2013 Meta-analysis reveals evolution in invasive plant species but little support for Evolution of Increased Competitive Ability (EICA). Ecol. Evol. **3**, 739-751. (10.1002/ece3.488) 23531703 PMC3605860

[86] Montesinos D, Santiago G, Callaway RM. 2012 Neo-allopatry and rapid reproductive isolation. Am. Nat. **180**, 529-533. (10.1086/667585) 22976015

[87] Ollerton J et al. 2019 The diversity and evolution of pollination systems in large plant clades: apocynaceae as a case study. Ann. Bot. **123**, 311-325. (10.1093/aob/mcy127) 30099492 PMC6344220

[88] Beavon MA, Kelly D. 2012 Invasional meltdown: pollination of the invasive liana *Passiflora tripartita* var. *mollissima* (Passifloraceae) in New Zealand. N. Z. J. Ecol. **36**, 100-107.

[89] Janzen DH. 1968 Reproductive behavior in the Passifloraceae and some of its pollinators in Central America. Behaviour **32**, 33-48. (10.1163/156853968X00072)

[90] Vieira MF, Leite M, Grossi JAS, Alvarenga EM. 2004 Reproductive biology of *Cryptostegia madagascariensis* Bojer ex Decne. (Periplocoideae, Apocynaceae), an ornamental and exotic species of Brazil. Bragantia **63**, 325-334. (10.1590/S0006-87052004000300002)

[91] Walther RU. 1995 Pollenfracht als Indikator für Ressourcennutzung und Einnischung bei madagassischen Schwärmern (Lepidoptera). PhD thesis, Friedrich–Alexander University, Erlangen, Germany.

[92] Watts S, Dormann CF, Martín González AM, Ollerton J. 2016 The influence of floral traits on specialization and modularity of plant–pollinator networks in a biodiversity hotspot in the Peruvian Andes. Ann. Bot. **118**, 415-429. (10.1093/aob/mcw114) 27562649 PMC4998976

[93] García M, Benítez-Vieyra S, Sérsic AN, Pauw A, Cocucci AA, Traveset A, Sazatornil F, Paiaro V. 2020 Is variation in flower shape and length among native and non-native populations of *Nicotiana glauca* a product of pollinator-mediated selection? Evol. Ecol. **34**, 893-913. (10.1007/s10682-020-10082-w)

[94] Hilton LD, Harder SC. 2006 Ecology and evolution of flowers. Oxford, UK: Oxford University Press.

[95] Murren CJ, Chang CC, Dudash MR. 2009 Patterns of selection of two North American native and nonnative populations of monkeyflower (Phrymaceae). New Phytol. **183**, 691-701. (10.1111/j.1469-8137.2009.02928.x) 19566813

[96] Stout JC, Duffy KJ, Egan PA, Harbourne M, Hodkinson TR. 2015 Genetic diversity and floral width variation in introduced and native populations of a long-lived woody perennial. AoB Plants **7**, plu087. (10.1093/aobpla/plu087) PMC4323518 25527475

[97] Egan PA, Stevenson PC, Tiedeken EJ, Wright GA, Boylan F, Stout JC. 2016 Plant toxin levels in nectar vary spatially across native and introduced populations. J. Ecol. **104**, 1106-1115. (10.1111/1365-2745.12573)

[98] Mackin CR, Peña JF, Blanco MA, Balfour NJ, Castellanos MC. 2021 Rapid evolution of a floral trait following acquisition of novel pollinators. J. Ecol. **109**, 2234-2246. (10.1111/1365-2745.13636)

[99] Jeschke JM, Pyšek P, Heger T. 2018 Tens rule. In Invasion biology: hypotheses and evidence, pp. 124-132. Wallingford, UK: CABI.

[100] Van Kleunen M, Manning JC, Pasqualetto V, Johnson SD. 2008 Phylogenetically independent associations between autonomous self-fertilization and plant invasiveness. Am. Nat. **171**, 195-201. (10.1086/525057) 18197772

[101] Ollerton J, McCollin D, Fautin DG, Allen GR. 2007 Finding NEMO: nestedness engendered by mutualistic organization in anemonefish and their hosts. Proc. R. Soc. B **274**, 591-598. (10.1098/rspb.2006.3758) PMC1766375 17476781

[102] Schofield PJ, Akins L. 2019 Non-native marine fishes in Florida: updated checklist, population status and early detection/rapid response. BioInvasions Record **8**, 898-910. (10.3391/bir.2019.8.4.18)

[103] Zenetos A, Apostolopoulos G, Crocetta F. 2016 Aquaria kept marine fish species possibly released in the Mediterranean Sea: first confirmation of intentional release in the wild. Acta Ichthyol. Piscat. **46**, 255-262. (10.3750/AIP2016.46.3.10)

[104] Mack RN, Simberloff D, Mark Lonsdale W, Evans H, Clout M, Bazzaz FA. 2000 Biotic invasions: causes, epidemiology, global consequences, and control. Ecol. Appl. **10**, 689-710. (10.1890/1051-0761(2000)010[0689:BICEGC]2.0.CO;2)

[105] Heimpel GE, Mills NJ. 2017 Biological control. Cambridge, UK: Cambridge University Press.

[106] Read D. 1998 The mycorrhizal status of *Pinus*. In Ecology and biogeography of *Pinus* (ed. DM Richardson). Cambridge, UK: Cambridge University Press.

[107] Schwartz MW, Hoeksema JD, Gehring CA, Johnson NC, Klironomos JN, Abbott LK, Pringle A. 2006 The promise and the potential consequences of the global transport of mycorrhizal fungal inoculum. Ecol. Lett. **9**, 501-515. (10.1111/j.1461-0248.2006.00910.x) 16643296

[108] Thiet RK, Boerner R. 2007 Spatial patterns of ectomycorrhizal fungal inoculum in arbuscular mycorrhizal barrens communities: implications for controlling invasion by *Pinus virginiana*. Mycorrhiza **17**, 507-517. (10.1007/s00572-007-0123-8) 17356853

[109] Barrett SC, Harder LD. 2017 The ecology of mating and its evolutionary consequences in seed plants. Ann. Rev. Ecol. Evol. Syst. **48**, 135-157. (10.1146/annurev-ecolsys-110316-023021)

[110] Redmond CM, Stout JC. 2018 Breeding system and pollination ecology of a potentially invasive alien *Clematis vitalba* L. in Ireland. J. Plant. Ecol. **11**, 56-63. (10.1093/jpe/trw137)

[111] Ferguson B, Lin M-H, Gresshoff PM. 2013 Regulation of legume nodulation by acidic growth conditions. Plant Signal. Behav. **8**, e23426. (10.4161/psb.23426) 23333963 PMC3676511

[112] Sheppard A, Hill R, DeClerck-Floate R, McClay A, Olckers T, Quimby P, Zimmermann H. 2003 A global review of risk-benefit-cost analysis for the introduction of classical biological control agents against weeds: a crisis in the making? Biocontrol News Information **24**, 91N-108N. (10.1079/bio20043008816)

[113] Diagne C, Leroy B, Vaissière A-C, Gozlan RE, Roiz D, Jarić I, Salles J-M, Bradshaw CJ, Courchamp F. 2021 High and rising economic costs of biological invasions worldwide. Nature **592**, 571-576. (10.1038/s41586-021-03405-6) 33790468

[114] Moles AT, Dalrymple RL, Raghu S, Bonser SP, Ollerton J. 2022 Data from: Advancing the missed mutualist hypothesis, the under-appreciated twin of the enemy release hypothesis. *Figshare*. (10.6084/m9.figshare.c.6214748) PMC9579764 36259169

